# Phage-Based Fluorescent Biosensor Prototypes to Specifically Detect Enteric Bacteria Such as *E*. *coli* and *Salmonella enterica* Typhimurium

**DOI:** 10.1371/journal.pone.0131466

**Published:** 2015-07-17

**Authors:** Manon Vinay, Nathalie Franche, Gérald Grégori, Jean-Raphaël Fantino, Flavie Pouillot, Mireille Ansaldi

**Affiliations:** 1 Laboratoire de Chimie Bactérienne, UMR7283, Centre National de la Recherche Scientifique, Aix-Marseille Université, Marseille, France; 2 Aix-Marseille Université, Université Sud Toulon Var, IRD, CNRS, Mediterranean Institute of Oceanology UM110, Marseille, France; 3 Pherecydes-Pharma, Romainville, France; Cairo University, EGYPT

## Abstract

Water safety is a major concern for public health and for natural environment preservation. We propose to use bacteriophages to develop biosensor tools able to detect human and animal pathogens present in water. For this purpose, we take advantage of the highly discriminating properties of the bacteriophages, which specifically infect their bacterial hosts. The challenge is to use a fluorescent reporter protein that will be synthesized, and thus detected, only once the specific recognition step between a genetically modified temperate bacteriophage and its bacterial host has occurred. To ensure the accuracy and the execution speed of our system, we developed a test that does not require bacterial growth, since a simple 1-hour infection step is required. To ensure a high sensitivity of our tool and in order to detect up to a single bacterium, fluorescence is measured using a portable flow cytometer, also allowing on-site detection. In this study, we have constructed and characterized several "phagosensor" prototypes using the HK620 bacteriophage and its host *Escherichia coli* TD2158 and we successfully adapted this method to *Salmonella* detection. We show that the method is fast, robust and sensitive, allowing the detection of as few as 10 bacteria per ml with no concentration nor enrichment step. Moreover, the test is functional in sea water and allows the detection of alive bacteria. Further development will aim to develop phagosensors adapted on demand to the detection of any human or animal pathogen that may be present in water.

## Introduction

Water quality is a major concern for public health and costal economics. It has a tremendous impact on infectious disease prevention, environment preservation and it is a financial burden for maritime activity and seaside resorts. Bacterial contaminations of bathing water are mostly responsible, for humans, of gastroenteritis, respiratory and mucosa infections [1]. The guideline for detection of microbiological contamination is described in the bathing water European directive 2006/7/CE [2]. Thus, a bathing water is considered as excellent when less than 250 *E*. *coli* and 100 intestinal enterococci are present in 100 ml of water. Current standards assess bacterial contamination as indicative of levels of fecal pollution that serves as a proxy for other infectious agents such as bacterial pathogens and viruses. However, detection of pathogenic bacteria in the absence of indicator of fecal pollution has been reported [3,4].

Procedures described by EU regulation are based on the counting of bacteria able to grow and to form a bacterial colony on selective media [2]. This conventional and laborious method is time-consuming (24 to 48 hours). Alternative methods are urgently needed to assess qualitatively and quantitatively the bacterial load of a given water sample. Among the methodologies developed nowadays, the quantitative polymerase chain reaction (qPCR) method constitutes a rapid tool to detect and quantify microorganisms. However, this technique requires sample preparation and must be performed in a laboratory by expert staff.

Since their discovery by F. D'Herelle and F. Twort [5,6], bacteriophages, the viruses that specifically infect bacteria, were intensively used in biotechnologies as therapeutic agents and molecular biology tools ([7–10] for reviews). Environmental bacteriophages provide a huge pool of specific biological devices to detect pathogenic bacteria. Indeed, to isolate a specific bacteriophage, one just need to sample water, originating from ponds, sewage or polluted seawater, and to proceed to a classical phage isolation and purification on the targeted bacterial strain [11,12]. Currently, several based-phage methods are developed to detect pathogenic bacteria using engineered phages expressing and coding for fluorescent [13,14], luminescent [15,16] or colorimetric [17] markers that require host cell infection for detection. Alternatively, labeled phage protein [18,19] and phage DNA staining [20,21] allow direct detection of phages bound to host bacterial cells. Several strategies have been developed for phage engineering such as *in vitro* packaging of modified DNA, transposition and post-infection homologous recombination [22–24]. However, time and sensitivity constraints to detect pathogenic bacteria support the quest to improve detection and identification of bacterial species in water.

In this study, we have designed and developed a rapid and highly sensitive phage-based biosensor, named phagosensor. For rapidity sake, this assay includes a single 1-hour infection step with a recombinant phage coupled to direct detection by flow cytometry that can be carried out on-site with an adequate compact and portable instrument. The engineering method is based on the lambda Red recombineering technique [25] performed on an integrated prophage, which can be thus easily manipulated as a part of the host genome. We developed our tool with a specific phage-host couple HK620-*E*. *coli* TD2158 [26,27], used as a prototype. We then successfully applied the method to *Salmonella* detection. This study constitutes a proof of concept that can be easily enlarged to detect various bacterial species.

## Materials and Methods

### Bacterial strains, bacteriophages and media used in this study

Bacterial strains and bacteriophages used in this study are listed in Table A in S1 File. The presence of the prophage was checked by PCR amplification of the *att*L site using dedicated primer pairs attL-ter/attL-pro (Table B in S1 File) [28]. When necessary ampicillin (100 μg.ml^-1^) or kanamycin (40 μg.ml^-1^) were added to growth media. M9 minimal medium supplemented with casamino acids (0.2%), D-glucose (0.3%), vitamin B1 (1 mg.ml^-1^), MgSO_4_ (0.2 mM), CaCl_2_ (0.1 mM) was used for fluorescence detection assays and LB medium was used to resuspend bacteria incubated in sea water samples.

### Phage engineering

**Fig 1:**
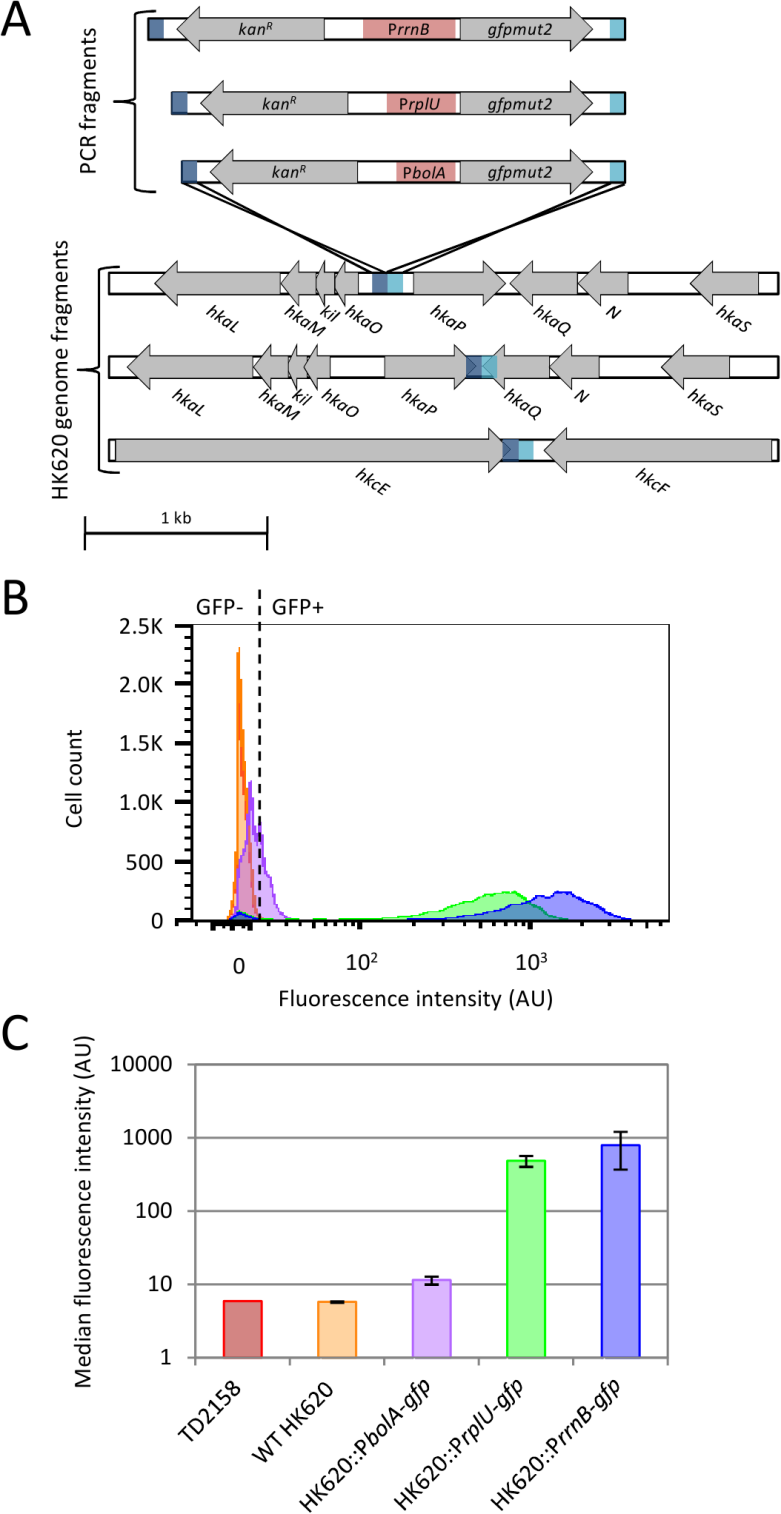
Characterisation of phagosensor constructs. (A) Three PCR fragments containing the *gfpmut2* gene under the control of either P*rrnB*, P*rplU* or P*bolA* promoter (red boxes), the kanamycin resistance-cassette and 80 bp homologous to the insertion region (dark and light blue) were recombined into the *hkaO*/*hkaP*, *hkaP*/*hkaQ* or *hkcE*/*hkcF* intergenic regions on the HK620 genome by the lambda Red recombination. (B) Fluorescence intensities of infected bacterial population measured by flow cytometry. Bacteria were infected for 1 hour at 37°C. Cytometry histograms represent the number of fluorescent or non fluorescent cells gated to the bacterial population as a function of the fluorescence intensity. The dotted line represents the limit between negative fluorescence (GFP-) and positive fluorescence (GFP+) determined by the fluorescent negative control WT HK620. Color code: non infected *E*. *coli* TD2158 (red), *E*. *coli* TD2158 infected with WT HK620 (orange) or recombinant phages HK620::P*bolA*-*gfp* (purple), HK620::P*rplU*-*gfp* (green), HK620::P*rrnB*-*gfp* (blue) (C) Histograms represent the means of median fluorescence intensities obtained with triplicate samples for 3 constructs. Color code is identical to (B).

Recombinant prophages were constructed by the insertion of *gfpmut2* [29] and kanamycin resistance genes into intergenic regions of HK620 and P22 prophages using the lambda Red homologous recombination method [25]. PCR fragments used as substrates for homologous recombination are described in Fig 1A. They contain the *gfpmut2* gene under the control of either P*rrnB*, P*rplU* or P*bolA* promoter and the kanamycin resistance-cassette, flanked by 80 bp of homology with the integration region. PCRs were performed using the high fidelity DNA polymerase PrimeSTAR Max Premix 2X (Takara), plasmids P*rrnB-gfp*, P*rplU-gfp*, P*bolA-gfp* (1 ng.μl^-1^) as templates and primer pairs listed in Table B in S1 File [30]. PCR fragments purified using the QIAquick PCR purification Kit (Qiagen) were treated with the restriction enzyme DpnI (Takara) in order to eliminate the template plasmids. After a purification step using the MinElute reaction cleanup kit (Qiagen), 1.5 μg of PCR fragments were electroporated into LCB6205(HK620)/pKD46 and LT2(P22)/pKD46 using the Gene PulserXcell electroporation system (Biorad). Fluorescent colonies were selected with a Fujifilm FLA-5100 scanner. The integration of the *gfpmut2* fragment in *hkaO*/*hkaP*, *hkaP*/*hkaQ*, *hkcE*/*hkcF* and *gp45*/*gp46* intergenic regions was checked by PCR using *hkaO*_dir/*hkaP*_R, *hkaP*_2_dir/*hkaQ*_dir, *hkcE*_dir/*hkcE*_down_rev, P22_*gp45*_dir/P22_*gp46*_dir, respectively. Two isolation rounds were performed to select for recombinant lysogenic bacteria only.

### Fluorescence counting by analytical flow cytometry

Following infection in M9 medium, cells were fixed with an equal volume of paraformaldehyde (PFA) 3.2% solution (Alfa Aesar) in PBS 1X (Euromedex). Samples were analyzed using the compact and highly sensitive flow cytometer A50-micro (Apogee Flow Systems, UK) equipped with an argon ion laser (Asbly, wavelength excitation 488 nm, 50 mV) and specific fluorescence filter set (Green (Gn): 535/35 nm, Orange (Or): 585/20 nm and Red (Rd): >610 nm). The sheath liquid was made of distilled water (filtered on 0.2 μm). Calibration beads (Apogee Flow Systems, 1 μm, excitation 488 nm and a broad fluorescence emission in the Gn, Or and Rd channels, 5,000 event.μl^-1^) were used as a standard every 10 samples to ensure quality control of the flow cytometer. Each sample was run in triplicate using the following settings: constant sample flow rate; sheath pressure 200 mbar; photodetector voltages for small and large angle light scatter, Gn, Or and Red fluorescences were respectively fixed at 200, 275, 400, 518 and 550 V. Depending on the analyses, the acquisition was triggered either on small and large angle light scatter with threshold values fixed at 362/65535 and 215/65535, or triggered on the Gn fluorescence (19/65535), respectively. Data were acquired in Log scale using PC control v3.40 and histogram v110.0 softwares (Apogee Flow Systems) but analyzed with FlowJoV10 software (TreeStarInc). The small versus large angle light scatter color plot was used to discriminate bacterial populations from the background noise. Then, fluorescence intensities gated on the bacterial populations were compared using an histogram that displays the number of bacteria per unit of fluorescence intensity. As far as fluorescence intensities are concerned, a bi-exponential representation was used to better display the bacteria. This display consists in a data transformation in order to use a linear scale to display low signals (intensities close to the origin) and a logarithmic scale for the larger ones (intensities from 10 to 10,000). This is a “Logicle” display method widely accepted by cytometrists that avoids deceptive effects of logarithmic scaling for low signals and compensated data in flow cytometry [31].The bacterial count in the non-fixed sample was calculated with the following formula: $\left(\frac{cell\;number}{debit\times time}\right)\times 2$.

### Optimal infection conditions

An overnight culture of *E*. *coli* TD2158 was diluted to a final cell count of 2x10^3^ bacteria per ml in supplemented M9 medium. This bacterial suspension was infected with the phage HK620::P*rrnB-gfp* then incubated either at 25°C, 30°C or 37°C. The bacterial count and the fluorescence intensities were analyzed by flow cytometry, as described above, immediately after addition of recombinant phage then each 30 min for 2 hours.

### Flow cytometry sensitivity assay

An overnight culture of *E*. *coli* TD2158 or *S*. *enterica* was diluted to a final cell count of 2x10^3^ bacteria per ml. Two-fold serial dilutions into M9 supplemented medium were prepared and 1 ml of each bacterial dilution was infected with 2x10^8^ PFU of either HK620::P*rrnB*-*gfp* or P22::P*rrnB-gfp*. After 1 hour at 30°C, PFA fixed sample were analyzed using flow cytometry. The green fluorescence was used as a trigger signal in order to detect the fluorescent population only. Cell count was calculated as above for each dilution.

### Immuno-labeling and epifluorescence microscopy

Overnight cultures of *E*. *coli* TD2158 and *Salmonella enterica* Typhimurium 14028S were mixed in a 1:10 ratio. The mixed sample infected with HK620::P*rrnB-gfp* was incubated at 30°C for 1 hour and analyzed by flow cytometry and epifluorescence microscopy. *S*. *enterica* bacteria were specifically labeled with phycoerythrin-coupled antibodies as follows. First, 500 μl of culture were peleted 10 min at 6,700 g and washed with 500 μl of PBS 1X containing NH_4_Cl 10 mM. Fifty μl of a suspension of anti-LPS from *S*. *enterica* Thyphimurium (IgG1 clone #1E6, Meridian Life Science; 6.4 μg.ml^-1^) were added to the bacterial suspension and incubated 20 min at room temperature. Then, 50 μl of a donkey anti-Mouse IgG conjugated with R-phycoerythrin (Jackson Immuno Research; 20 μg.ml^-1^) were added and incubated for 10 min at room temperature in the dark. Epifluorescence microscopy was performed using an inverted epifluorescence microscope (Nikon TiE-PFS) coupled to a CCD camera (Hamamatsu OrcaR2). A 100x NA1.3 oil PhC objective and adequate filter set (Semrock HQ TxRed and Semrock HQ GFP) were used to detect red and green fluorescent bacteria. Bacterial suspensions (5 μl) were transferred on a microscope slide and covered with an agarose pad (1.5% in M9 1X). Data were analyzed using the ImageJ software [32].

### Detection in sea water

Three water samples were taken from Saint-Cyr sur Mer coastal area (GPS coordinates, X: 43.183331 Y: 5.71667). No specific permissions were required for water sampling in public coastal area located outside a national park and the field studies did not involve endangered or protected species. Non-sterilized water samples, naturally containing marine organisms, were artificially contaminated with 2x10^6^ *E*. *coli* TD2158 cells.ml^-1^ in stationary phase then incubated 20 h at 20°C under shaking. The spiked sea water samples were then grossly filtered (cellulose filters, 30 μm) to remove any particles bigger than 30 μm that could interfere with flow cytometry, but bacterial cells were not removed. Bacterial cells (10 ml) were peleted by centrifugation at 4,500 g for 30 min and resuspended in 10 ml LB medium to allow bacterial metabolism to resume and thus GPF production. Immediately after the resuspension step, 1 ml of sample was infected with 2x10^8^ PFU of HK620::P*rrnB-gfp*, incubated for 1 h at 30°C, fixed in 3.2% of PFA, and then analyzed by flow cytometry as described above. The limit of bacterial detection was calculated using two-fold serial dilutions of the bacterial culture into LB medium followed by HK620::P*rrnB*-*gfp* infection. Data were acquired in Log scale using PC control v3.40 and histogram v110.0 softwares (Apogee Flow Systems) and analyzed with FlowJoV10 software (TreeStarInc). The trend line is obtained from the mean of normalized values (1,000 bacteria.ml^-1^ starting cell count).

## Results

### Design and construction of 9 phagosensor constructs based on the HK620 temperate phage

HK620 phagosensors were designed to fit the following requirements: *(i)* express the *gfpmut2* gene at a high level to allow non ambiguous fluorescence detection of bacteria in mixed samples; *(ii)* produce fluorescence only in infected cells; *(iii)* integrate PCR fragments by lambda Red recombination without disturbing the phage lytic and lysogenic cycles [25]. We designed several constructs in order to choose the best prototype for further experiments. Briefly, PCR fragments containing the *gfpmut2* gene under the control of three different promoters (P*rrnB*, P*rplU* and P*bolA*) associated to the divergently oriented *kan* ^*R*^ cassette and flanked by 80 bp of sequence homology with the target sequences (Fig 1A) were electroporated into LCB6205(HK620) lysogenic strain carrying plasmid pKD46 [25]. The promoter sequences (Table B in S1 File) were chosen to obtain the *gfp* gene under the control of a stationary phase specific promoter (P*bolA*), and of two strong σ70 promoters (P*rplU* and P*rrnB*), the later being the strongest [33]. Target regions for homologous recombination were chosen to avoid the disruption of any transcriptional unit or promoter region. Three regions were chosen, one consists of an intergenic region between two divergent genes (*hkaO*/*hkaP*), the remaining two consist of regions between convergent genes (*hkaP*/*hkaQ* and *hkcE*/*hkcF*) (Fig 1A) [27]. Seven constructs out of nine attempted were obtained (Table A in S1 File). All 3 promoter regions were successfully recombined into the *hkcE*/*hkcF* region, however, despite several attempts, the construct containing the P*rrnB* promoter could not be obtained in the *hkaO*/*hkaP* and *hkaP*/*hkaQ* regions.

### Induction and viability of engineered phages

All phage constructs were tested for mitomycin C (MMC) induction, to make sure they were functional and used for further infection and particle titration, to check their viability. All tested constructs behaved as the wild-type phage (data not shown) and post-MMC induction titers were around 1x10^10^ pfu.ml^-1^ for all of them. Constructs in the *hkcE*/*hkcF* region, namely HK620::P*bolA-gfp*, HK620::P*rplU-gfp*, and HK620::P*rrnB-gfp* were chosen for further characterization.

### Characterization of various phagosensor constructs

We first looked at *gfp* expression and fluorescence emission during lysogenic growth. The engineered phages showed typical lysogenic growth when added at a MOI = 100 to the HK620 host *E*. *coli* TD2158 strain. Compared to the growth curve obtained in the absence of any phage, the lysogenic curves show a period of growth of around 2 hours, followed by a lysis step and a re-growth phase of the lysogens after 4 hours of infection (Fig A in S1 File, upper left panel). The same infected cultures were monitored for fluorescence emission and although all phage constructs emitted fluorescence, timing and levels were very different (Fig A in S1 File, upper right panel). The P*bolA-gfp* construct was not as efficient in GFP production as the two other constructs that showed high fluorescence emission as soon as 1 hour and up to 5 hours post-infection. Maxima of fluorescence intensities were obtained after 4 hours of infection in all cases. This maximum was 3-fold and 46.5-fold higher with the P*rrnB-gfp* construct than those measured with P*rplU-gfp* and P*bolA-gfp*, respectively (Fig A in S1 File, upper right panel). Moreover, only 50 min were necessary to obtain a fluorescence intensity of 500 AU when the *gfp* gene was under the control of the P*rrnB* promoter versus 1 hour 15 min and 3 hours 25 min with P*rplU* and P*bolA* promoters, respectively.

The results obtained by flow cytometry confirmed these data. The fluorescence emission of the culture infected with P*bolA-gfp* was almost indistinguishable from the experiment conducted with the WT phage (Fig 1B). In contrast, a vast majority of the population of infected cells with either HK620::P*rplU-gfp* or HK620::P*rrnB-gfp* produced GFP at a high level. However, the population infected with HK620::P*rrnB-gfp* showed significantly higher fluorescence intensities than with HK620::P*rplU-gfp* (Fig 1B and 1C). Since the fluorescence emission was stable and homogenous for more than five hours with HK620::P*rrnB-gfp* (Fig A in S1 File), this engineered phage was chosen for further experiments.

We then assayed the viability and fluorescence production upon bacterial infection of the engineered phages conserved as indicated in the Materials and Methods section over a one year period. Two different assays were performed periodically: *(i)* phage titration, and *(ii)* bacterial infection and fluorescence detection of the infected population (Fig A in S1 File, bottom). Both HK620::P*rplU-gfp*, and HK620::P*rrnB-gfp* constructs proved to conserve constant titers when conserved at 4°C in Tris-HCl 50 mM pH 8.0, NaCl 100 mM. Moreover, the fluorescence measured after infection of the host bacteria with the phage preparations after one year was comparable to the one measured just after phage production and purification for the two constructs analyzed (Fig A in S1 File, bottom). These results show that viability and ability of infection were not affected by the engineering method.

### Choosing optimal infection conditions

**Fig 2:**
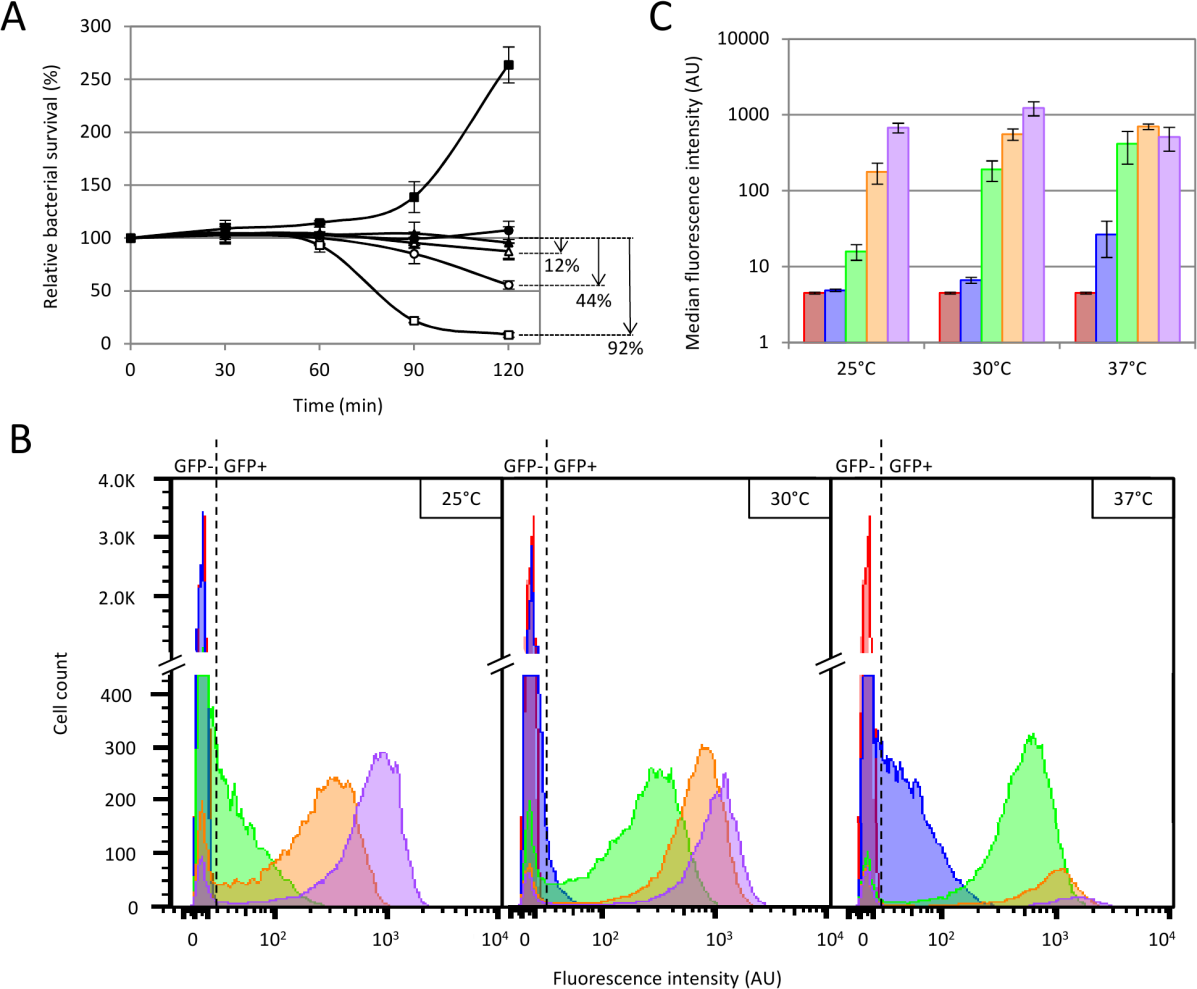
Determination of optimal detection conditions. (A) Bacterial survival analyzed by flow cytometry as a function of time under several temperature conditions. Bacterial cell counts were measured immediately after infection then each 30 min for 2 hours. *E*. *coli* TD2158 was either non infected (dark symbols) or infected with HK620::P*rrnB*-*gfp* phage (white symbols). Cultures were incubated at 25°C (triangles), 30°C (circles), or 37°C (squares). The relative bacterial survival is the ratio between the bacterial cell count at 30 min, 60 min, 90 min, or 120 min and the initial bacterial cell count multiplied by 100. Arrows show the percentage of lysed bacteria 2 hours post-infection. (B) and (C) Fluorescence intensity obtained with phagosensors under several temperature conditions. *E*. *coli* TD2158 was infected with HK620::P*rrnB*-*gfp* phage and incubated at 25°C, 30°C or 37°C. Fluorescence intensities were monitored immediately after infection (red) then 30 min (blue), 60 min (green), 90 min (orange) and 120 min (purple) post-infection. (B) Cytometry histograms represent the number of fluorescent or non-fluorescent cells gated to the bacterial population as a function of the fluorescence intensity. Panels from left to right correspond to results obtained at 25°C, 30°C and 37°C, respectively. The dotted line represents the limit between negative fluorescence (GFP-) and positive fluorescence (GFP+) determined by using the fluorescent negative control WT HK620. (C) Column histograms represent the means of median fluorescence intensities obtained with a triplicate of sample incubated at 25°C, 30°C or 37°C.

Optimal infection conditions should not allow post-infection bacterial growth or lysis. Thus, we assayed population survival under several temperature conditions (Fig 2A and Table C in S1 File). It was obvious that 37°C was the worst condition since host lysis occurred at a high frequency past 60 min reaching 92% of lysed cells at 120 min. Both 25°C and 30°C conditions displayed lower lysis profiles, in particular at 60 min, where 100% of the host cells were still intact (Fig 2A, filled circles and triangles). We next checked fluorescence emission at the single cell level by flow cytometry as a function of time (0, 30, 60, 90, 120 min post-infection) and under different temperature conditions (25, 30 and 37°C) (Fig 2B and 2C). The results evidenced that fluorescence intensities measured 30 min post-infection under all temperature conditions did not segregate from the control intensities (no phage or infection with a WT phage). The same observation was made at 25°C 60 min post-infection with HK620::P*rrnB-gfp* (Fig 2B). At 60 min post-infection and at 37°C, the measured fluorescence intensities were high but concerned only a small fraction of cells, as a result of cell lysis observed at this temperature (Fig 2). In contrast, infections carried out at 30°C produced high fluorescence intensities and concerned a large and homogenous fraction of cells since lysis was limited at this very temperature. Together, these results indicate that infection at 30°C for 60 min, constitutes the best conditions to use this biosensor, since host cell lysis was inexistent, and that the medians of measured fluorescence upon infection were high enough to allow a robust discrimination from the control experiments.

### What is the limit of detection?

**Fig 3:**
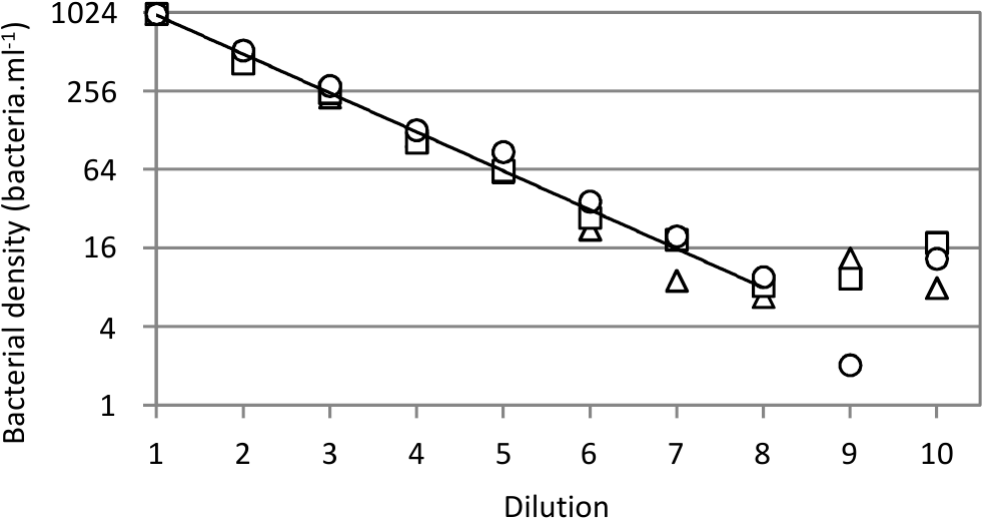
Determination of minimal detectable cell count of infected *E*. *coli* TD2158. Serial 2-fold dilutions from 10^3^ to 2 bacteria.ml^-1^ are numbered from 1 to 10. Each dilution was infected with the HK620::P*rrnB*-*gfp* phage and incubated for 1 hour at 30°C. The bacterial cell count was determined using the A50 Apogee flow cytometer. This assay was repeated three times and each replicate is represented with the following symbols: triangle, replicate 1; square, replicate 2; circle, replicate 3. The trend line obtained from the mean of normalized values (1,000 cells.ml^-1^ starting cell count) is drawn on the chart. The correlation coefficients (dilutions from 1 to 8) were 0.993, 0.995 and 0.997 for replicates 1, 2 and 3, respectively.

Samples containing 10^3^ bacteria.ml^-1^ were serially 2-fold diluted in M9 supplemented medium before infection with HK620::P*rrnB-gfp* at 30°C. At 60 min post-infection, each sample was fixed with PFA and analyzed by flow cytometry. In order to focus on the fluorescent population only, the detection settings were modified to allow a correct discrimination of the fluorescent bacteria from the autofluorescence of the medium (see the Materials and Methods section). The results obtained on triplicate samples (Fig 3) show a very good correlation (R^2^>0.993 for all three replicates) for cell counts down to less than 10 cells per ml. This assay is thus able to detect very diluted bacteria through fluorescence detection.

### Detection of stationary phase bacteria in sea water

**Fig 4:**
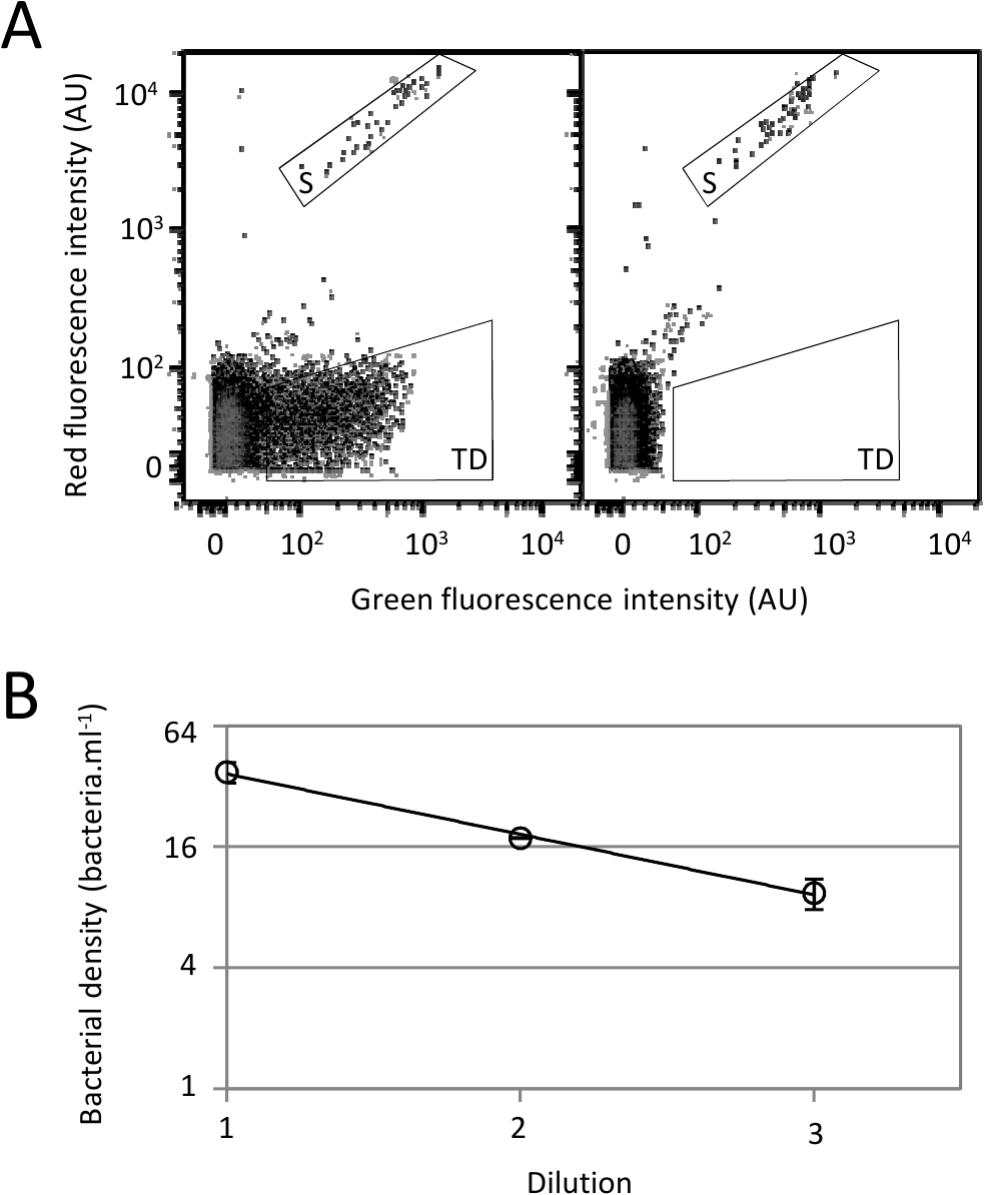
Flow cytometric analysis of spiked sea water sample. *E*. *coli* TD2158 in stationary phase was added to a sea water sample to a final cell count of 2x10^6^ bacteria.ml^-1^, then the picked sea water sample was incubated overnight at 20°C. (A) Samples infected with HK620::P*rrnB-gfp* (left cytogram) or non infected (right cytogram) were analyzed using flow cytometer. *E*. *coli* TD2158 (TD) and *Synechococcus* (S) populations were discriminated by dot-plots of red fluorescence intensity versus green fluorescence intensity. (B) Serial 2-fold dilutions of spiked sea water was infected with the HK620::P*rrnB-gfp* phage and incubated for 1 hour at 30°C. The bacterial cell count was determined using flow cytometry.

Because the weaker metabolic activity of bacteria under starving conditions could alter the infectivity and the induced signal [34,35], we tested the detection of *E*. *coli* TD2158 in non sterilized and non-filtered sea water samples. Furthermore, the photosynthetic plankton naturally present in sea water emits autofluorescence and could provoke false positive results. Water samples were loaded with 2x10^6^ bacteria.ml^-1^ then they were incubated at 20°C for 20 h to mimic contamination by enteric bacteria. Thus, *E*. *coli* TD2158 cells should be in the same metabolic state than Enterobacteria present during an accidental contamination of water. Under such conditions, Enterobacteria such as TD2158 should not grow. To test that, we spiked a sea water sample with fluorescent bacteria (LCB6139) and measured the number of cells by flow cytometry before and after incubation in sea water for 20 h at 20°C. We observed that for an initial bacterial load of 4.98 x 10^6^ bacteria.ml^-1^, the cell count measured after incubation was 2.93 x 10^6^ bacteria.ml^-1^. Therefore, the cells did not divide under such conditions, but around 50% are rather dying, probably due to predators (protozoa) naturally occurring in non treated sea water samples. Consequently, for each experiment, we measured the number of cfu after incubation, and these numbers were set as references for the dilution assays. Fig 4A shows that infected bacteria emitted strong green fluorescence intensity after resuspension into LB medium and infection at 30°C for 1 h. The region representing the target population was designated TD. By contrast, no bacteria was present in the TD region either when cells were not infected with HK620::P*rrnB-gfp* or when non spiked sample was infected with phages (Fig 4A). These controls confirmed that the detection was specific to *E*. *coli* TD2158 cells infected with HK620::P*rrnB-gfp*. Moreover, the multiparametric data obtained by using flow cytometry allowed to discriminate green fluorescent bacteria from autofluorescent bacteria present in sea water such as *Synechococcus*. This microorganism presents a specific fluorescence profile due to its pigment content, essentially chlorophyll and phycoerythrin, which emits red and orange fluorescence, respectively. Due to overlapping spectra, orange fluorescence was detected using the green fluorescence channel allowing *Synechococcus* population detection on dot-plots of red fluorescence intensity versus green fluorescence intensity (Fig 4A). Under starving conditions, reactivation of the metabolism was slower, and therefore spiked samples had to be resuspended in rich medium (see Materials and Methods). Flow cytometry analysis decreased the false positive risk because naturally autofluorescent bacteria were discriminated from target bacteria. The detection limit into an artificially contaminated sample was determined by serial two-fold dilution analysis at 10 cells per ml as obtained previously (Fig 4B).

### Specificity of the engineered phage

**Fig 5:**
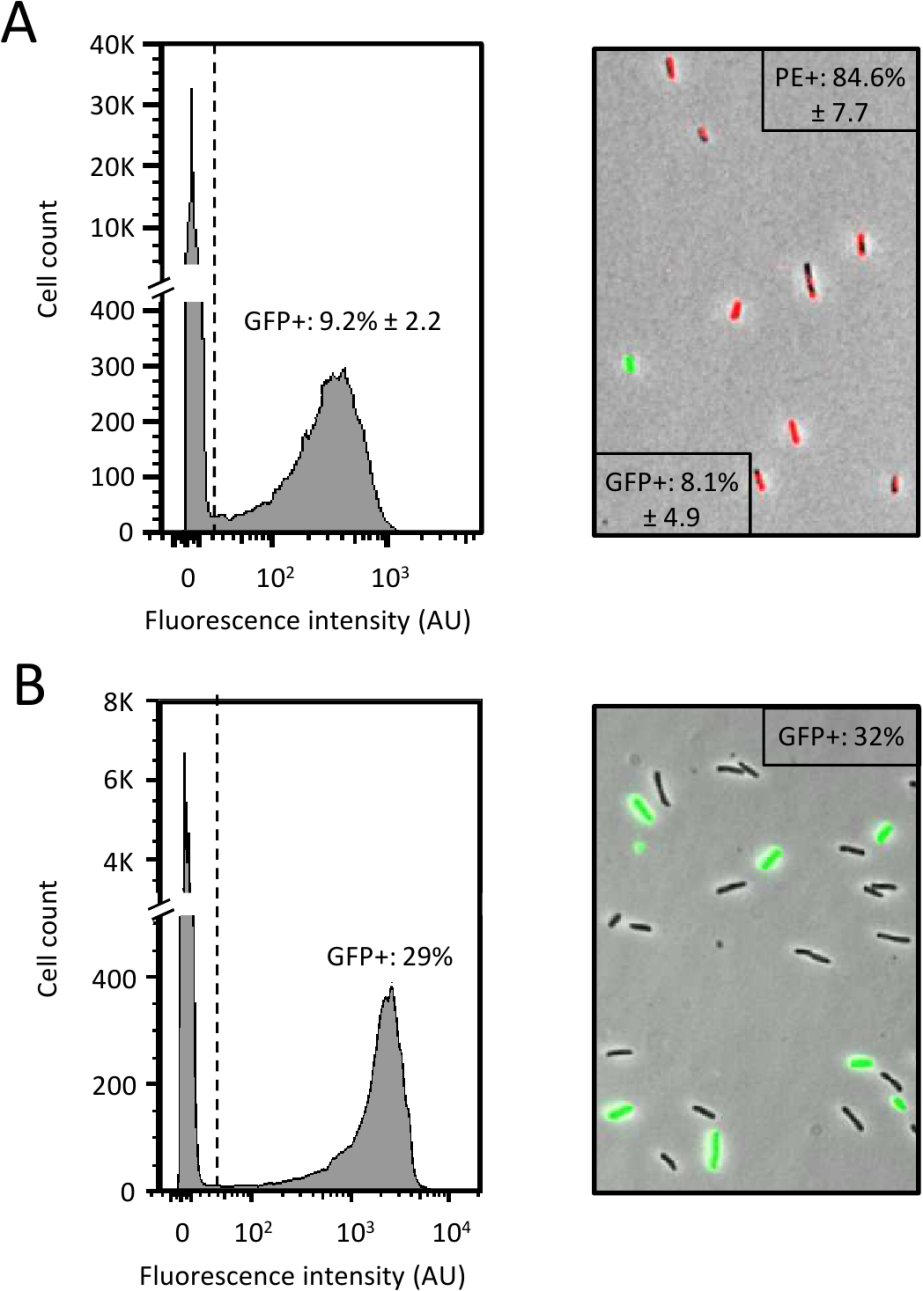
Specific detection of *E*. *coli* TD2158 with the phagosensor HK620::PrrnB-gfp. Co-cultures of *E*. *coli* TD2158 and *S*. *enterica* (ratio = 1:10) (A) or *E*. *coli* TD2158 and *E*. *coli* MG1655 (ratio 3:10) (B), were infected with the HK620::P*rrnB*-*gfp* phage and incubated at 30°C for 1 hour. The fluorescence intensity of individual cells gated to the bacterial population was determined by flow cytometry (left panels). The dotted line represents the limit between negative (no fluorescence) and positive (fluorescence) cells as determined using the fluorescence negative control composed of a non infected co-culture. Mean fluorescence was calculated from at least triplicate samples. Co-culture samples were analyzed by epifluorescence microscopy imaging of co-culture sample (Right panels). Red fluorescence population was *S*. *enterica* labeled with antibody coupled with phycoerythrin (PE), GFP-induced green fluorescent population was the infected bacteria and non fluorescent bacteria were E. coli MG1655. Average fluorescence values were obtained from triplicate samples.

To pursue with the characterization of the engineered HK620 phage we needed to assay the specific detection of *E*. *coli* TD2158 cells in a mix of different bacterial strains. An *E*. *coli* TD2158 suspension at 2x10^6^ cells.ml^-1^ was mixed with *S*. *enterica* Typhimurium 14028S suspension at 2x10^7^ cells.ml^-1^ (ratio 1:10). The mixed suspension was infected with the HK620::P*rrnB-gfp* construct at 30°C for one hour followed by flow cytometry analysis. Alternatively, the mix was stained with labeled antibodies that specifically detect *S*. *enterica* cells and analyzed by microscopy. As shown in Fig 5A, the HK620::P*rrnB-gfp* biosensor detected 9.2% of *E*. *coli* TD2158 cells in the bacterial mix, in agreement with the initial ratio of 1:10 (*E*. *coli* TD2158: *S*. *enterica*). A similar result was obtained with fluorescence microscopy (Fig 5A, 8.1% of *E*. *coli* TD2158). The slight discrepancy between the two analyses can be easily explained by the higher robustness of the flow cytometry method. Indeed, a few hundred of cells were analyzed by microscopy whereas several thousands of cells were counted using flow cytometry. The same experiment was realized using *E*. *coli* TD2158 cells mixed to *E*. *coli* MG1655 (ratio 1:3). Results showed that HK620::P*rrnB-gfp* detected specifically *E*. *coli* TD2158 in agreement with the initial ratio of 1:3 (Fig 5B, 29% of *E*. *coli* TD2158). Together, these results show that our biosensor prototype is able to quantitatively discriminate between two types of closely related cells, even when the target cells constitute a minority in a given mixture.

### Detection of *Salmonella enterica* Typhimurium

**Fig 6:**
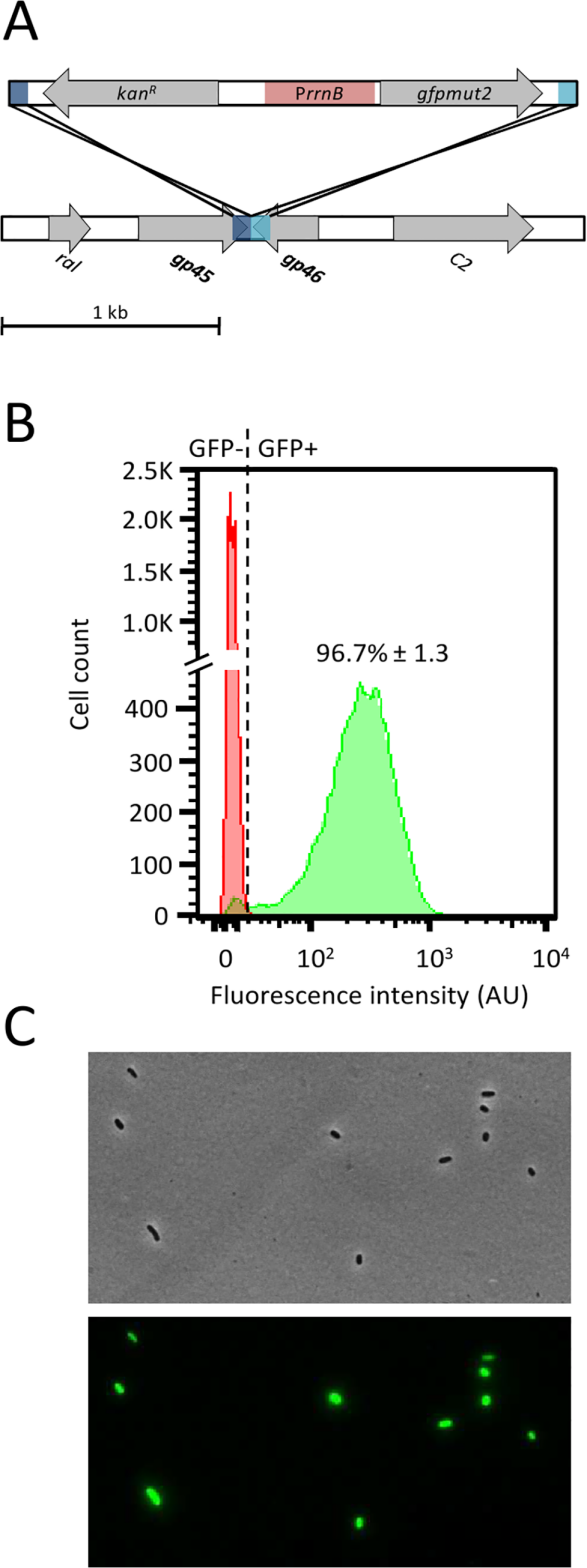
Specific detection of *Salmonella enterica* Typhimurium LT2 with a P22 phagosensor. (A) Schematic representation of the P22 construct. A PCR fragment containing the *gfpmut2* gene under the control of P*rrnB* promoter (red boxes), the kanamycin resistance-cassette and 80 bp homologous to the insertion region (dark and light blue) was recombined into the P22 *gp45/gp46* intergenic region. (B) A culture of *S*. *enterica* Typhimurium LT2 was either infected with the P22::P*rrnB*-*gfp* phage (green curve) or non infected (red curve) then incubated at 30°C for 1 hour. Average fluorescent cells number was obtained from triplicate samples. (C) Optical (upper) and fluorescence (lower) microscope images of *S*. *enterica* Typhimurium LT2 incubated at 30°C for 1 h with P22::P*rrnB*-*gfp*.

The aim of this study was to be able to apply this method to the detection of pathogenic bacteria. Based on the results obtained with the HK620 prototype, we easily constructed a recombinant P22 phage in which the *gfpmut2* gene was integrated in the *gp45*/*gp46* intergenic region (Fig 6A). Recombinant phage was propagated on LT2 with the same efficiency as the WT P22 (data not shown). A stationary phase culture of LT2 cells was infected with P22::P*rrnB-gfp* at 30°C for 1 h, such as determined with the HK620 prototype. Under these conditions, the fluorescence intensity of infected bacterial population was similar to that observed with HK620 and high enough to allow an accurate discrimination of fluorescent bacteria (Fig 6B). Under such conditions, the P22::P*rrnB-gfp* biosensor was able to detect 96.7% of LT2 cells. First, these results demonstrate that the prototype method can be rapidly applied to construct an efficient phagosensor to detect *Salmonella enterica* Typhimurium. Second, the infection conditions developed with the HK620::P*rrnB-gfp* prototype was successfully transposed to *S*. Thyphimurium detection.

## Discussion

Bacteriophages have been intensively used as molecular biology tools since their discovery [9]. In the last decade, several phage-based bioassays have been developed using a variety of output signals such as fluorescence and luminescence and based on direct (phage protein fusions or phage DNA staining) [19–21] or indirect (phages labeled with antibodies or quantum dots) detection [18]. Most techniques require a high level of technicality as well as dedicated detection device that are rarely transportable on the sampling site. We chose to use fluorescence as an output signal and flow cytometry as a rapid and real time detection device because when used in combination they allow both high sensitivity and, with an adequate instrument, portability. To achieve this latter goal we have used a robust, compact, and portable flow cytometer that was initially developed for the US army to detect pathogen spores in deserts (Desert Storm operation) and that is currently widely used for HIV field detection campaigns in Africa [36,37]. This flow cytometer is indeed optimized for small particle thanks to a special device developed by H. Steen to optimize the light scattering of viral and other submicroscopic particles [38].

Most phage-based biosensors use capsid gene fusions that allow bacterial detection upon binding of the labeled phage to its bacterial receptor [18,19,21]. This is a source of false positive, since metabolically inactive bacteria are detected together with metabolically active ones. By using promoter based *gfp* fusions, we avoid this phenomenon since detection occurs only after the phage genome injection step and only by metabolically active cells able to produce the GFP protein. Even cells with a slow metabolism can be detected this way, since stationary phase bacteria, just diluted into fresh minimal medium, are detected before growth resumes since we observed no change in cell count at 30°C for at least 90 minutes (Fig 2A). Moreover, methods using small capsid gene fusion are dependent on the phage nature. Phage burst size differ from one phage to another, and in order to obtain significant signals, one has to adapt the copy number of the reporter gene [39]. In this study, we demonstrate that having the reporter gene under the control of a strong bacterial promoter allow to obtain similar detection characteristics even thought two different phages were used (HK620 and P22) (Figs 1 and 6). The main improvement of the present method is the high sensitivity that is achieved. We were able to specifically and reproducibly detect as little as 10 bacterial cells.ml^-1^ in supplemented M9 medium, without any concentration step (Fig 3). The most sensitive methods to date using phage as biosensor without concentration or enrichment steps are detecting between 10^2^ to 10^4^ bacterial cells per ml [40]. This is an important achievement because this method can detect enterobacterial loads within the same order of magnitude of those mentioned in the European directive of microbiological contamination [2]. Another important feature of this assay is its rapid implementation. Indeed, from sampling to detection, the limitation is the incubation time to allow phage infection and GFP production. However, without compromising the detection level, we were able to set up a reproducible and standard infection time of 1 hour at 30°C (Fig 2). Since no additional treatment is needed, fluorescence is then directly analyzed by flow cytometry, and results can be obtained within minutes.

The biosensor prototype was developed with a highly specific phage that infects the environmental *E*. *coli* TD2158 strain [26]. As a result, the assay we developed allows a specific detection of this strain even in diluted samples or in mixed co-cultures (Figs 3 and 4). Based on these results, it is thus possible to design specific biosensor tools to detect any given bacterial strain, as long as the engineering methods is available for this strain [41–46] or wide range detectors if several specific phagosensors are mixed into the same cocktail. Thus, this technique enlarges the possibility of detection and identification of bacteria in diluted samples.

## Supporting Information

S1 File Supporting Information. Strains and bacteriophages used in this study (Table A). Plasmids and primer pairs used in this study (Table B). Bacterial survival rate as a function of time under several temperature conditions (Table C). **Fig A. Fluorescence emission upon infection with engineered HK620 phages.** Upper panels, bacterial growth and fluorescence profile obtained from non infected *E*. *coli* TD2158 alone (red), infected with WT HK620 (orange) or recombinant phages HK620::P*bolA-gfp* (purple), HK620::P*rplU-gfp* (green), HK620::P*rrnB-gfp* (blue) were compared. Top panels represent growth curves (top left panel) and culture fluorescence (right panel) obtained using a microplate reader. Bacterial growth, lytic and lysogenic growth phases are indicated by 1, 2 and 3, respectively. Fluorescence intensity was determined using the equation: (sample fluorescence – medium autofluorescence) / OD_600_. Bottom panel, engineered phages conservation was studied over 1 year by measuring phages titers and post-infection fluorescence intensities. Column histograms represent fluorescence intensities obtained after 1 hour incubation at 37°C of *E*. *coli* TD2158 infected with WT HK620 (orange) or recombinant phages HK620::P*rplU-gfp* (green), HK620::P*rrnB-gfp* (blue). Fluorescence intensity was obtained using a microplate reader and determined with the equation: (sample fluorescence – medium autofluorescence) / OD_600_. Stability of phages during several weeks is represented by titration curves of WT and recombinant HK620 phages.
(DOCX)
