# Supplementary material for: Phage-Based Fluorescent Biosensor Prototypes to Specifically Detect Enteric Bacteria Such as E. coli and Salmonella enterica Typhimurium
Source: PLoS One. 2015 Jul 17;10(7):e0131466. doi: 10.1371/journal.pone.0131466 (PMC4506075; doi:10.1371/journal.pone.0131466)
Supplement: S1 File — Strains and bacteriophages used in this study (Table A). Plasmids and primer pairs used in this study (Table B). Bacterial survival rate as a function of time under several temperature conditions (Table C). Fig A. Fluorescence emission upon infection with engineered HK620 phages. Upper panels, bacterial growth and fluorescence profile obtained from non infected E. coli TD2158 alone (red), infected with WT HK620 (orange) or recombinant phages HK620::PbolA-gfp (purple), HK620::PrplU-gfp (green), HK620::PrrnB-gfp (blue) were compared. Top panels represent growth curves (top left panel) and culture fluorescence (right panel) obtained using a microplate reader. Bacterial growth, lytic and lysogenic growth phases are indicated by 1, 2 and 3, respectively. Fluorescence intensity was determined using the equation: (sample fluorescence – medium autofluorescence) / OD600. Bottom panel, engineered phages conservation was studied over 1 year by measuring phages titers and post-infection fluorescence intensities. Column histograms represent fluorescence intensities obtained after 1 hour incubation at 37°C of E. coli TD2158 infected with WT HK620 (orange) or recombinant phages HK620::PrplU-gfp (green), HK620::PrrnB-gfp (blue). Fluorescence intensity was obtained using a microplate reader and determined with the equation: (sample fluorescence – medium autofluorescence) / OD600. Stability of phages during several weeks is represented by titration curves of WT and recombinant HK620 phages. (DOCX) [file pone.0131466.s001.docx]

# Additional methods, Figure and Tables.

# Phage-based fluorescent biosensor prototypes to specifically detect enteric bacteria such as *E.* *coli* and *Salmonella* *enterica* Typhimurium

Manon Vinay,^1^ Nathalie Franche,^1^ Gérald Grégori,^2^ Jean-Raphaël Fantino,^1^ Flavie Pouillot,^3^ Mireille Ansaldi ^1^#

Laboratoire de Chimie Bactérienne, UMR7283, Centre National de la Recherche Scientifique, Aix-Marseille Université, Marseille, France ^1^; Aix-Marseille Université, Université Sud Toulon Var, IRD, CNRS, Mediterranean Institute of Oceanology UM110, Marseille, France ^2^; Pherecydes-Pharma, Romainville, France ^3^

Running Head: Phagosensor prototypes, conception and application.

# Address correspondence to Mireille Ansaldi, mireille.ansaldi@imm.cnrs.fr

# Additional methods.

## MMC induction, production and purification of recombinant phages.

Recombinant phages were collected following MMC induction (5 µg.ml^-1^) under shaking for 3 hours. Then, cells were treated twice with chloroform and peleted by centrifugation at 4,500 g for 10 min, the supernatants were filtered (0.22 µm) and stocked at 4°C. For phage production, 100 µl of phage suspension (around 10^9^ PFU.ml^-1^) and 100 µl of an overnight host culture were mixed with 3 ml of LB soft agar and spread onto a LB plate. After incubation overnight at 37°C, the top agar layer was collected and mixed with 10 ml of LB. The mix was left standing for 30 min to allow phage diffusion into the medium and treated twice with chloroform as described above. Phages were then purified by centrifugation at 20,000 g for 1 hour. Phage pellets were resuspended in Tris-HCl 50 mM pH 8.0, NaCl 100 mM, buffer and incubated overnight at 4°C under shaking. Purified phage suspensions were tittered and kept at 4°C.

## Phage conservation*.*

Over a whole year, we have regularly measured the activity of phages kept at 4°C. Test infections were performed using an overnight culture of *E. coli* TD2158 and whole population fluorescence was recorded as described below using a microplate reader. Infections with WT and recombinant HK620 phages (P*rplU-gfp* and P*rrnB-gfp*) were performed with a constant volume (50 µl) of phage solutions.

## Whole population fluorescence detection using a microplate reader*.*

All assays were performed in triplicate in 96-well black microplates with clear bottom (Greiner) on infected bacterial cultures. Values of OD_600_ and fluorescence (excitation wavelength: 488 nm; emission wavelength: 521 nm, integration time 20 µs, gain 100 and 25 reads per sample) of bacterial suspensions were monitored every 10 min for 12 h using a microplate reader Infinite M200 (TECAN). Values represent the averages of 3 independent samples. The fluorescence intensities were calculated as follows: the fluorescence value of blank (M9 supplemented medium) was subtracted to the fluorescence value of the samples and divided by the OD_600_ of the samples (sample fluorescence – medium autofluorescence) / OD_600_.

## Table A. Strains and bacteriophages used in this study

| **Strain** | **Characteristics** | **Reference** |
| --- | --- | --- |
| *E. coli* TD2158 | Environmental *Escherichia coli* H strain | [1] |
| LCB6205 | Phage-free *Escherichia coli* TD2158, previously named *E. coli* TD2158_PL4 | [2] |
| LCB6205(HK620) | LCB6205 lysogen for HK620 | This work |
| LCB6133 | LCB6205(HK620 *hkaOP*::P*bolA-gfp*), Km^R^ | This work |
| LCB6134 | LCB6205(HK620 *hkaOP*::P*rplU-gfp*), Km^R^ | This work |
| LCB6142 | LCB6205(HK620 *hkaPQ*::P*bolA-gfp*), Km^R^ | This work |
| LCB6143 | LCB6205(HK620 *hkaPQ*::P*rplU-gfp*), Km^R^ | This work |
| LCB6138 | LCB6205(HK620 *hkcEF*::P*bolA-gfp*), Km^R^ | This work |
| LCB6140 | LCB6205(HK620 *hkcEF*::P*rplU-gfp*), Km^R^ | This work |
| LCB6139 | LCB6205(HK620 *hkcEF*::P*rrnB-gfp*), Km^R^ | This work |
| *Salmonella enterica* Typhimurium ATCC 14028 | A strain derived from CDC 60-6516 | L. Bossi collection, Molecular Genetic Center, CNRS |
| LT2 | *S.* *enterica* Typhimurium WT strain | A. Aertsen collection, Laboratory of Food Microbiology, KU Leuven |
| LT2(P22) | *S. enterica Typhimurium* LT2 lysogen for P22 phage | [3] |
| LCB6219 | LT2(P22 *gp45-46*::P*rrnB-gfp*), Km^R^ | This work |
| **Bacteriophage** |  |  |
| HK620 | Temperate bacteriophage | [1] |
| HK620::P*bolA-gfp* | Recombinant phage induced from LCB6138 | This work |
| HK620::P*rplU-gfp* | Recombinant phage induced from LCB6140 | This work |
| HK620::P*rrnB-gfp* | Recombinant phage induced from LCB6139 | This work |
| P22 | Temperate bacteriophage | A. Aertsen collection, Laboratory of Food Microbiology, KU Leuven |
| P22::P*rrnB-gfp* | Recombinant phage induced from LCB6219 | This work |

## Table B. Plasmids and primer pairs used in this study

| **Plasmid** | **Characteristics** | **Reference** |
| --- | --- | --- |
| pKD46 | lambda Red expression plasmid. *bla*, *araC*, *gam*, *bet*, *exo* genes; Pbad promoter ; ori R101 | [4] |
| P*rrnB-gfp* | pUA66 containing the P*rrnB* promoter region | [5] |
| P*bolA-gfp* | pUA66 containing the P*bolA* promoter region | [5] |
| P*rplU-gfp* | pUA139 containing the P*rplU* promoter region | E. Bouveret |
| **Primer** |  |  |
| attL-ter | CATCGAGAAGGCGGTATGGTTTTTC | [6] |
| attL-pro | AATGGATATAACGAGCCCCTCC | [6] |
| kan_bsq4 ^a^ | TCACATTGGAGGGCAAAGAAGATTTCCAATAATCAGAACAAGTCGGCTCCTGTTTAGTTACGAGCGACATTGCTCCGTGT**ATCCAGATGGAGTTCTGAGGTC** |  |
| gfpmut2_bsq4 ^a^ | GAGAACCACCGAGCCTGATGTGGTTAAAAGACAGGCATACGAATAAACACTGCACTGTGTATTCATTCCAATGAGTGAAT**CGTTCACCGACAAACAACAG** |  |
| hkaO_dir | TCAAGTAAAGATTCGGAAGG |  |
| hkaP_R | CATAATTCTCAGCTTGCACA |  |
| kan_hkaPQ ^a^ | ATAGAGCGCCAGACCCTAAGCCTGTATATGTACCGCCGCTGACAAATTATTAATTTTATTCCTGAAATGGCCTCTTCACC**ATCCAGATGGAGTTCTGAGGTC** |  |
| gfpmut2_hkaPQ ^a^ | TTCAACTTTACATCGACTTAAGGAAAGTATTAAGGCGAATGGTCAAAACTGAAGGAGATATCCCCCTGACAAAGTAAGGG**CGTTCACCGACAAACAACAG** |  |
| hkaP_2_dir | TGTATATGTACCGCCGCTGA |  |
| hkaQ_dir | CATTACTGCTTGTCAGGCAAA |  |
| kan_hkcEF ^a^ | GTTCAGTTAGTGTGAGGTTGATGCTGCAGCGTCAGGTGTGAGTATTTGATATCTACGAAATAATGTGCTTTTGCATAATT**ATCCAGATGGAGTTCTGAGGTC** |  |
| gfpmut2_hkcEF ^a^ | AATATCAATTACATCTTTCTGTTTTATGGTGTGACCATTACATGCTTTTATATAAACAATTCTTCTTGGTTTATCATGTA**CGTTCACCGACAAACAACAG** |  |
| hkcE_dir | TTGGTTGGGGTTGGGTATAA |  |
| hkcE_down_rev | CACCACGAGGTGGTTAAAGC |  |
| kan_P22gp45/46^a^ | tgagatttgccgggaagtcagacattcttatgaatgaacttattgtacaggacgaacagctcaaaaaataacgacttaac**ATCCAGATGGAGTTCTGAGGTC** |  |
| gfpmut2_P22gp45/46^a^ | gtacttcttccaggcgtagcactttacgcggctggtcatcgtaagagcaaacaaataacagcgaggtaaggtatttgtcg**CGTTCACCGACAAACAACAG** |  |
| P22_gp45_dir | ATCACTCGATTGCCTTCTGC |  |
| P22_gp46_dir | GCCAAAAGAATTGACCGTGT |  |

^a^: sequences in bold match onto pUA66 derived plasmids

## Table C. Bacterial survival rate as a function of time under several temperature conditions

|  | TD2158 + HK620::P*rrnB-gfp* | | | TD2158 | | |
| --- | --- | --- | --- | --- | --- | --- |
|  | 25°C | 30°C | 37°C | 25°C | 30°C | 37°C |
| 0 min | **100**^a^ | **100**^a^ | **100**^a^ | 100 | 100 | 100 |
| 30 min | **104**^a^ | **102**^a^ | **105**^a^ | 104 | 103 | 109 |
| 60 min | **104**^a^ | **100**^a^ | 93 | 103 | 103 | 114 |
| 90 min | 96 | 85 | 22 | 104 | 99 | 139 |
| 120 min | 88 | 56 | 8 | 96 | 107 | 264 |

^a^: numbers in bold correspond to infected samples where bacterial lysis was not observed.

**
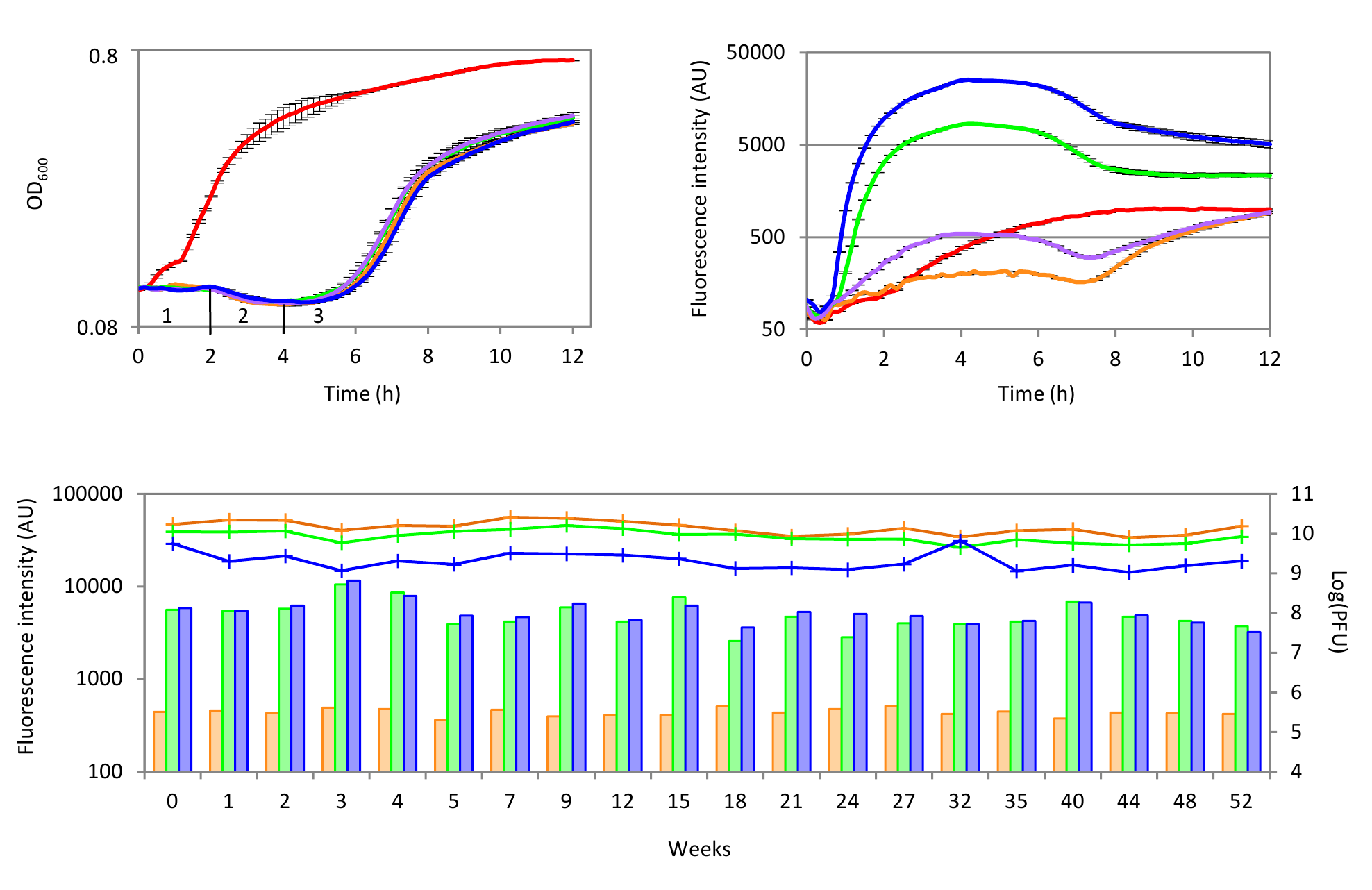
**

Figure A: Fluorescence emission upon infection with engineered HK620 phages.

Upper panels, bacterial growth and fluorescence profile obtained from non infected *E. coli* TD2158 alone (red), infected with WT HK620 (orange) or recombinant phages HK620::P*bolA-gfp* (purple), HK620::P*rplU-gfp* (green), HK620::P*rrnB-gfp* (blue) were compared. Top panels represent growth curves (top left panel) and culture fluorescence (right panel) obtained using a microplate reader. Bacterial growth, lytic and lysogenic growth phases are indicated by 1, 2 and 3, respectively. Fluorescence intensity was determined using the equation: (sample fluorescence – medium autofluorescence) / OD_600_. Bottom panel, engineered phages conservation was studied over 1 year by measuring phages titers and post-infection fluorescence intensities. Column histograms represent fluorescence intensities obtained after 1 hour incubation at 37°C of *E. coli* TD2158 infected with WT HK620 (orange) or recombinant phages HK620::P*rplU-gfp* (green), HK620::P*rrnB-gfp* (blue). Fluorescence intensity was obtained using a microplate reader and determined with the equation: (sample fluorescence – medium autofluorescence) / OD_600_. Stability of phages during several weeks is represented by titration curves of WT and recombinant HK620 phages.

1. Dhillon TS, Poon AP., Chan D, Clark AJ (1998) General transducing phages like *Salmonella* phage P22 isolated using a smooth strain of *Escherichia coli* as host. FEMS Microbiol Lett 161: 129–133. doi:10.1111/j.1574-6968.1998.tb12938.x.

2. Menouni R, Champ S, Espinosa L, Boudvillain M, Ansaldi M (2013) Transcription termination controls prophage maintenance in *Escherichia coli* genomes. Proc Natl Acad Sci U S A 110: 14414–14419. doi:10.1073/pnas.1303400110.

3. Cenens W, Mebrhatu MT, Makumi A, Ceyssens P-J, Lavigne R, et al. (2013) Expression of a Novel P22 ORFan Gene Reveals the Phage Carrier State in *Salmonella Typhimurium*. PLoS Genet 9: e1003269. doi:10.1371/journal.pgen.1003269.

4. Datsenko KA, Wanner BL (2000) One-step inactivation of chromosomal genes in *Escherichia coli* K-12 using PCR products. Proc Natl Acad Sci U S A 97: 6640–6645.

5. Zaslaver A, Bren A, Ronen M, Itzkovitz S, Kikoin I, et al. (2006) A comprehensive library of fluorescent transcriptional reporters for *Escherichia coli*. Nat Methods 3: 623–628. doi:10.1038/nmeth895.

6. Panis G, Duverger Y, Champ S, Ansaldi M (2010) Protein binding sites involved in the assembly of the KplE1 prophage intasome. Virology 404: 41–50.
